# Security Analysis of Continuous-Variable Measurement-Device-Independent Quantum Key Distribution Systems in Complex Communication Environments

**DOI:** 10.3390/e24010127

**Published:** 2022-01-14

**Authors:** Yi Zheng, Haobin Shi, Wei Pan, Quantao Wang, Jiahui Mao

**Affiliations:** School of Computer Science, Northwestern Polytechnical University, Xi’an 710129, China; shihaobin@nwpu.edu.cn (H.S.); panweihh@163.com (W.P.); wqt@nwpu.mail.edu.cn (Q.W.); maojiahui@nwpu.mail.edu.cn (J.M.)

**Keywords:** continuous-variable, quantum key distribution, measure-device-independent, fluctuating channel transmittance, security analysis

## Abstract

Continuous-variable measure-device-independent quantum key distribution (CV-MDI QKD) is proposed to remove all imperfections originating from detection. However, there are still some inevitable imperfections in a practical CV-MDI QKD system. For example, there is a fluctuating channel transmittance in the complex communication environments. Here we investigate the security of the system under the effects of the fluctuating channel transmittance, where the transmittance is regarded as a fixed value related to communication distance in theory. We first discuss the parameter estimation in fluctuating channel transmittance based on these establishing of channel models, which has an obvious deviation compared with the estimated parameters in the ideal case. Then, we show the evaluated results when the channel transmittance respectively obeys the two-point distribution and the uniform distribution. In particular, the two distributions can be easily realized under the manipulation of eavesdroppers. Finally, we analyze the secret key rate of the system when the channel transmittance obeys the above distributions. The simulation analysis indicates that a slight fluctuation of the channel transmittance may seriously reduce the performance of the system, especially in the extreme asymmetric case. Furthermore, the communication between Alice, Bob and Charlie may be immediately interrupted. Therefore, eavesdroppers can manipulate the channel transmittance to complete a denial-of-service attack in a practical CV-MDI QKD system. To resist this attack, the Gaussian post-selection method can be exploited to calibrate the parameter estimation to reduce the deterioration of performance of the system.

## 1. Introduction

Quantum key distribution (QKD) offers an unconditionally secure communication scheme to establish secret keys between the sender Alice and the receiver Bob through an insecure quantum channel in the presence of potential eavesdropper Eve, where the two remote partners are authenticated [1,2,3,4,5]. The security of the scheme is guaranteed by the basic laws of quantum mechanics [6,7,8]. At present, there are two kinds of QKD protocols: discrete-variable quantum key distribution (DVQKD) and continuous-variable quantum key distribution (CVQKD). In particular, CVQKD scheme based on the Gaussian-modulated coherent states (GMCS) can be well compatible with the classical optical communication systems, which has been fully proven to be secure against general attacks (e.g., the collective and coherent attacks) based on some ideal assumptions [8,9,10,11,12]. It has been experimentally implemented by many research groups in laboratories and in field environments [13,14,15,16,17,18]. In addition, the system has also been optimized by researchers from different aspects [19,20,21,22,23,24,25]. However, practical security problems seriously hinder the commercial development of CVQKD, where this obstacle is caused by the security loopholes opened by the gaps between the theoretical model and the practical system because the behavior of real devices typically deviates from that considered in the security proofs [26,27]. This problem also limits the application of DVQKD, which has been investigated by many researchers [28,29,30].

In a practical CVQKD system, Eve can exploit the above imperfections to successfully obtain secret key information without being detected, which is an effective quantum hacking strategy. For example, Eve can control the transmitted local oscillator (LO) to perform the LO fluctuation attack [31], LO calibration attack [32], and wavelength attack [33,34]. In addition, the imperfect linearity of homodyne detector can be exploited by Eve to launch saturation attack [35] and homodyne detector blinding attack [36]. Apart from this, laser damage attack against optical attenuator and laser seeding attack in light source have been proposed [37,38,39,40,41,42]. The security loopholes involved by these attacks can be closed by the corresponding countermeasures, which makes the system complicated. Moreover, there are some unknown attacks in practical CVQKD systems, which cannot be effectively resisted by the above schemes. Therefore, the researchers propose the continuous-variable measure-device-independent quantum key distribution (CV-MDI QKD) protocol to close all loopholes opened by imperfect detection [43,44,45,46,47,48,49,50,51,52,53]. In CV-MDI QKD, the measurement is performed by an untrusted third party, which is immune to all quantum hacking on detection. The research of CV-MDI QKD can promote the application of CVQKD.

According to the framework of CV-MDI QKD, the source and channel become the final battlefield between the authorized communication parties and Eve. Recently, the imperfections on source in practical CV-MDI QKD systems have been gradually researched [54,55,56]. In particular, the channel transmittance in theoretical model is considered to be a fixed value, which can be acquired based on the communication distance. However, practical communication environments are complex, which may result in the time-varying transmittance. In this work, we investigate the effects of the fluctuating channel transmittance for the security of practical CV-MDI QKD systems. Specifically, CV-MDI QKD in fluctuating channel transmittance is first described. Based on the model, we then show the difference of parameter estimation between this case and the stable channel case. To clearly quantify this difference, we discuss the specific parameter estimation when the channel transmittance respectively obeys the two-point distribution and the uniform distribution. Here, Eve can easily manipulate the channel to make the transmittance obey the above distributions. Subsequently, we analyze the secret key rate of the system based on the estimated parameter in different channel distributions. We observe that the fluctuating channel transmittance make the performance of the system deteriorated obviously, which may make communication interrupted. This impact is even greater in the extreme asymmetric case. These analyses indicate that the channel transmittance can be easily manipulated by Eve to launch a denial-service attack in a practical CV-MDI QKD system, which is different from the quantum hacking attack originating from security loopholes. Finally, the Gaussian post-selection technology can be exploited to calibrate the estimated parameters to prevent this attack.

The paper is organized as follows. In Section 2, parameter estimation in complex communication environments is shown for a practical CV-MDI QKD system, where these two theoretical channel models are established. Then, based on these models, we analyze the security of the system in the fluctuating channel transmittance when the channel transmittance respectively obeys the two-point distribution and the uniform distribution in Section 3. Finally, conclusions are presented in Section 4.

## 2. Channel Models and Parameter Estimation in Complex Communication Environments

**Figure 1:**
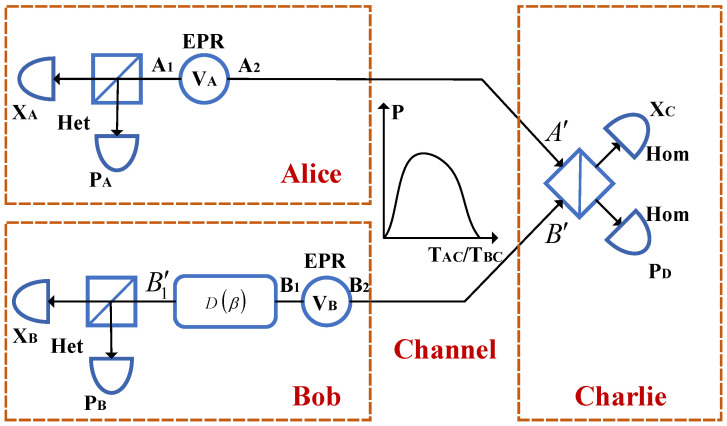
EB model of a practical GMCS CV-MDI-QKD system running in complex environments. Here, channel transmittance $T_{AC}$ and $T_{BC}$ are modeled to obey a certain distribution, which may be easily controlled by Eve.

Figure 1 shows the entanglement-based (EB) model of a GMCS CV-MDI-QKD protocol, which is fully equivalent to the standard prepare and measure (PM) model [45,46]. It is important to note that this equivalence is the core of security proofs for GMCS CVQKD protocols. In the EB model, one two-mode squeezed state with variance $V_{A}+1\left(V_{B}+1\right)$ is first prepared by Alice (Bob), where the mode $A_{1}\left(B_{1}\right)$ is measured by a heterodyne detector and the other mode $A_{2}\left(B_{2}\right)$ is sent to an unauthenticated third party, Charlie, through the quantum channel. The channel distance between Alice (Bob) and Charlie is $L_{AC}\left(L_{BC}\right)$, and the total transmission distance $L_{AB}$ should be $L_{AC}+L_{BC}$. Subsequently, Charlie interferes the received modes $A^{\prime }$ and $B^{\prime }$ at a beam splitter (BS) and obtains two output modes *C* and *D*. Then, two homodyne detectors are exploited by Charlie to measure the quadrature variable $x_{C}$ of mode *C* and quadrature variable $p_{D}$ of mode *D*, and the detection results $x_{C},\;p_{D}$ are immediately announced through a public channel. Finally, the mode $B_{1}$ is modified to $B_{1}^{\prime }$ by Bob through displacement operation D(β). Here $\beta =g_{m}\left(x_{C}+ip_{D}\right)$, and $g_{m}$ indicates the gain of the displacement operation. It is believed that the mode $A_{1}$ and $B_{1}^{\prime }$ become entangled after through these above steps. Therefore, Alice and Bob will share a group correlated vectors $X=\{\left(x_{A,i},x_{B,i}\right)|i=1,2,...,N\}$ or $P=\{\left(p_{A,i},p_{B,i}\right)|i=1,2,...,N\}$. These data can be used to estimate the channel transmittance $T_{AC}\left(T_{BC}\right)$ and the excess noise $\varepsilon_{AC}\left(\varepsilon_{BC}\right)$. In addition, key reconciliation and privacy amplification are exploited to further guarantee the security of the system.

According to the above analysis, there are two quantum channels in a practical CV-MDI-QKD system, i.e., $C_{AC}$ and $C_{BC}$, which are assumed to be a normal linear model with the following relations:

(1) $$\begin{array}{cc} \; & x_{A^{\prime }}=t_{AC}x_{A}+z_{AC},\\ \; & p_{A^{\prime }}=t_{AC}p_{A}+z_{AC},\\ \; & x_{B^{\prime }}=t_{BC}x_{B}+z_{BC},\\ \; & p_{B^{\prime }}=t_{BC}p_{B}+z_{BC}, \end{array}$$

where $x_{A}\left(p_{A}\right)$, $x_{A^{\prime }}\left(p_{A^{\prime }}\right)$, $x_{B}\left(p_{B}\right)$ and $x_{B^{\prime }}\left(p_{B^{\prime }}\right)$ represent the corresponding quadrature variables of the mode $A_{2}$, $A^{\prime }$, $B_{2}$ and $B^{\prime }$, $t_{AC}=\sqrt{T_{AC}}$, $t_{BC}=\sqrt{T_{BC}}$, $z_{AC}$ and $z_{BC}$ indicate the total noises in the aforementioned quantum channels. Here, $z_{AC}$ and $z_{BC}$ respectively obey two centered normal distributions with variance $\sigma_{AC}^{2}=T_{AC}\xi_{AC}+N_{0}$ and $\sigma_{BC}^{2}=T_{BC}\xi_{BC}+N_{0}$, where $\xi_{AC}=\varepsilon_{AC}N_{0}$, $\xi_{BC}=\varepsilon_{BC}N_{0}$, and $N_{0}$ is the shot-noise variance. Therefore, $t_{AC}$ and $\sigma_{AC}^{2}$ can be calculated as

(2) $$\begin{array}{cc} t_{AC} & =\frac{E(x_{A}x_{A^{\prime }})}{E(x_{A}^{2})},\\ \sigma_{AC}^{2} & =E[{\left(x_{A^{\prime }}-t_{AC}x_{A}\right)}^{2}]. \end{array}$$

It is no doubt that $t_{AC}$ and $\sigma_{AC}^{2}$ can also be acquired using $p_{A}$ and $p_{A^{\prime }}$. In addition, $t_{BC}$ and $\sigma_{BC}^{2}$ can be similarly calculated. In the following analysis, we only discuss the relevant calculation about channel $C_{AC}$. Based on the Equations (1) and (2), $T_{AC}$ and $\varepsilon_{AC}$ can be expressed by

(3) $$\begin{array}{cc} T_{AC} & =t_{AC}^{2}={\left[\frac{E(x_{A}x_{A^{\prime }})}{E(x_{A}^{2})}\right]}^{2},\\ \varepsilon_{AC} & =\frac{\sigma_{AC}^{2}-N_{0}}{N_{0}t_{AC}^{2}}\\ \; & =\frac{E\left[{\left(x_{A^{\prime }}-t_{AC}x_{A}\right)}^{2}\right]-N_{0}}{N_{0}T_{AC}}. \end{array}$$

In security proofs, the channel transmittance is assumed to be stable. Therefore, it is reasonably regarded as a fixed value related to transmission distance. However, practical communication environments are complex, which may result in a time-varying transmittance. In particular, the potential Eve may control the channel transmittance. To analyze the effects of the deviation, based on the phase space, $x_{A}$ and $x_{A}^{\prime }$ can be written as

(4) $$\begin{array}{cc} x_{A} & =|\alpha_{A}|\cos\theta_{A},\\ x_{A^{\prime }} & =\sqrt{T_{AC}}\left\{\right|\alpha_{A}\left|\cos\left(\theta_{A}+\Delta \varphi \right)+x_{\varepsilon_{AC}}\right\}+x_{N_{0}}, \end{array}$$

where $|\alpha_{A}|$ is the amplitude of the coherent states prepared by Alice, $\theta_{A}$ is the phase of these states, Δφ is the phase shift caused by complex channel environments. In particular, $x_{\varepsilon_{AC}}$ and $x_{N_{0}}$ are the additional values of quadratures variable $x_{A}$, which are caused by the channel excess noise $\varepsilon_{AC}$ and shot-noise $N_{0}$, respectively. We can further obtain

(5) $$\begin{array}{cc} E(x_{A}x_{A^{\prime }}) & =E\left(\sqrt{T_{AC}}{|\alpha_{A}|}^{2}\cos^{2}\theta_{A}\right)=E\left(\sqrt{T_{AC}}\right)V_{x_{A}},\\ E(x_{A}^{2}) & =E\left({|\alpha_{A}|}^{2}\cos^{2}\theta_{A}\right)=V_{x_{A}},\\ E(x_{A^{\prime }}^{2}) & =V_{x_{A}}E\left(T_{AC}\right)+\xi_{AC}E\left(T_{AC}\right)+N_{0}, \end{array}$$

where $V_{x_{A}}=V_{A}N_{0}$, $V_{A}$ is the modulation variance at Alice’s side. It is important to note that $T_{AC}$, $|\alpha_{A}|\cos\theta_{A}$, $N_{0}$ and $\xi_{AC}$ are totally independent. In addition, it is reasonable that Δφ is approximated to zero in the above analyses, because the phase noise can be extremely constrained by the high-precision phase compensation technique. Eventually, based on Equations (3) and (5), the estimated channel parameters ${\hat{T}}_{AC}$ and ${\hat{\varepsilon }}_{AC}$ in fluctuating channel transmittance should satisfy

(6) $$\begin{array}{cc} {\hat{T}}_{AC} & ={\left[\frac{E(x_{A}x_{A^{\prime }})}{E(x_{A}^{2})}\right]}^{2}={\left[E\left(\sqrt{T_{AC}}\right)\right]}^{2},\\ {\hat{\varepsilon }}_{AC} & =\frac{V_{A}E\left(T_{AC}\right)+\varepsilon_{AC}E\left(T_{AC}\right)-V_{A}{\left[E\left(\sqrt{T_{AC}}\right)\right]}^{2}}{{\left[E\left(\sqrt{T_{AC}}\right)\right]}^{2}}. \end{array}$$

Similarly, the estimated channel parameters ${\hat{T}}_{BC}$ and ${\hat{\varepsilon }}_{BC}$ in fluctuating channel transmittance also obey the above relations. There are some clear deviations between the estimated channel parameters in fluctuating channel transmittance and ideal values, which is closely related to the distribution of the fluctuating channel transmittance. Therefore, we need to quantify the distribution to analyze the effects of the fluctuating channel transmittance. However, the channel transmittance may irregularly change, which cannot be described using a specific formula. In particular, Eve may actively control the channel to disturb the transmittance. According to Ref. [57], the channel transmittance may be easily manipulated by Eve to obey the two-point distribution or the uniform distribution. Then, we discuss the estimated channel parameters when the channel transmittance obeys the two distributions.

**Figure 2:**
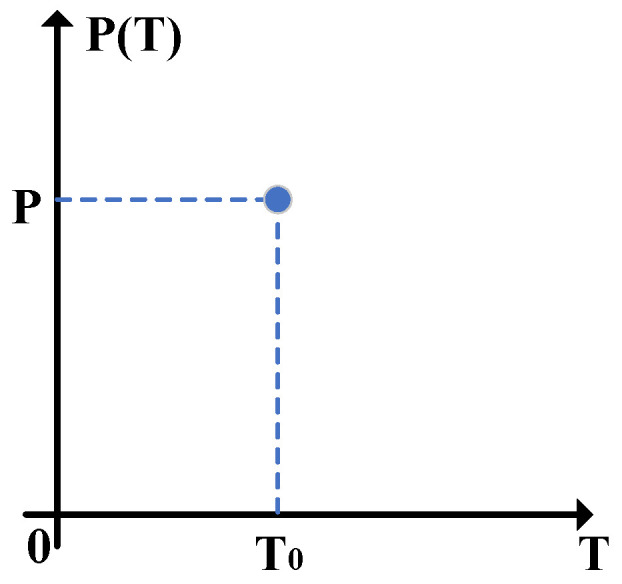
The probability density function of the channel transmittance when it obeys the two-point distribution, where *T* represents $T_{AC}$ or $T_{BC}$.

Figure 2 describes the probability density function when the channel transmittance obeys the two-point distribution, where the channel transmittance can vary between 0 and $T_{0}$ under the control of Eve. Therefore, $T_{AC}/T_{0}∼\left(1,P\right)$, where $T_{0}=10^{-0.02L_{AC}}$ represents the ideal channel transmittance and $L_{AC}$ is the transmission distance between Alice and Charlie. Correspondingly, we can obtain $E\left(T_{AC}\right)=PT_{0}$, $E\left(\sqrt{T_{AC}}\right)=P\sqrt{T_{0}}$. Eventually, based on Equation (6), the channel parameters can be evaluated as

(7) $${\hat{T}}_{AC,1}=P^{2}T_{0},\;{\hat{\varepsilon }}_{AC,1}=\frac{1}{P}V_{A}-V_{A}+\frac{1}{P}\varepsilon_{AC},$$

where *P* is the probability when the channel transmittance $T_{AC}$ equals to $T_{0}$, $\varepsilon_{AC}$ is the true channel excess noise, the number 1 indicates the two-point distribution. It is no doubt that the estimated channel parameters ${\hat{T}}_{BC,1}$ and ${\hat{\varepsilon }}_{BC,1}$ also satisfy Equation (7).

**Figure 3:**
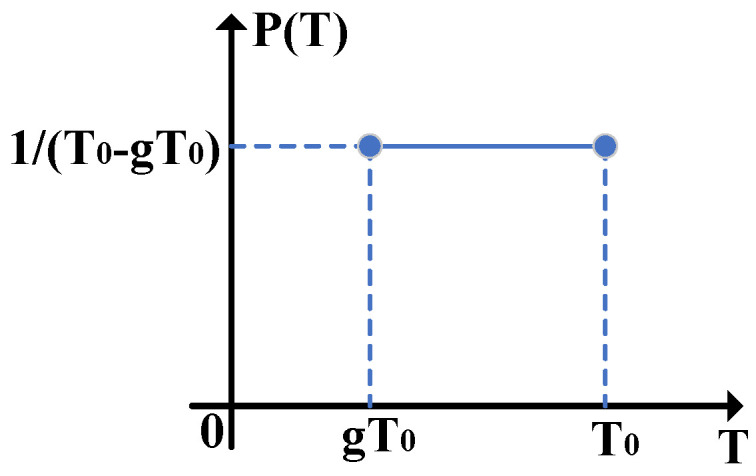
The probability density function of the channel transmittance when it obeys the uniform distribution, where *T* represents $T_{AC}$ or $T_{BC}$.

Figure 3 shows the probability density function of the channel transmittance when it obeys the uniform distribution. Here, $T_{AC}$ is a uniform distributed random number between $gT_{0}\left(0<g<1\right)$ and $T_{0}$, i.e., $T_{AC}∼U\left(gT_{0},T_{0}\right)$, where $T_{0}$ also represents the ideal channel transmittance. Therefore, $E(T_{AC})$ and $E(\sqrt{T_{AC}})$ can be calculated as

(8) $$\begin{array}{cc} E(T_{AC}) & =\int_{gT_{0}}^{T_{0}}\frac{1}{T_{0}-gT_{0}}T_{AC}dT_{AC}\\ \; & =\frac{\left(1+g\right)T_{0}}{2},\\ E(\sqrt{T_{AC}}) & =\int_{gT_{0}}^{T_{0}}\frac{1}{T_{0}-gT_{0}}\sqrt{T_{AC}}dT_{AC}\\ \; & =\frac{2\left(1-g^{\frac{3}{2}}\right)\sqrt{T_{0}}}{3(1-g)}. \end{array}$$

According to Equations (6) and (8), the estimated values of the channel parameters can be expressed as

(9) $$\begin{array}{cc} \; & {\hat{T}}_{AC,2}=\frac{4{\left(1-g^{\frac{3}{2}}\right)}^{2}T_{0}}{9{\left(1-g\right)}^{2}},\\ \; & {\hat{\varepsilon }}_{AC,2}=\\ \; & \frac{\left(1-9g+16g^{\frac{3}{2}}-9g^{2}+g^{3}\right)V_{A}+9\left(1-g-g^{2}+g^{3}\right)\varepsilon_{AC}}{8{\left(1-g^{\frac{3}{2}}\right)}^{2}}, \end{array}$$

where $\varepsilon_{AC}$ also represents the true excess noise, the number 2 indicates the uniform distribution. Similarly, ${\hat{T}}_{BC,2}$ and ${\hat{\varepsilon }}_{BC,2}$ also obey Equation (9). In the following analysis, the two-point and uniform distributions are considered to be common channel distribution models to investigate the effects of the fluctuating channel transmittance.

In addition, fiber dispersion and imperfect polarization compensation in a practical system may affect the accuracy of measurement, which makes the estimated channel parameters deviate from the practical values. Therefore, these imperfections can indirectly lead to the fluctuation of the channel transmittance. Here, this variation may be not regular, which is difficulty expressed by a mathematical formula. However, according to the above analysis, Eve may actively control channel to disturb the communication environments. She can easily manipulate the channel to make it obeys the above distributions. To facilitate security analysis, the two-point distribution and the uniform distribution can be considered to be common channel distribution models, which does not affect our conclusion.

## 3. Security Analysis

Secret key rate is a key parameter for the security and performance of a practical CV-MDI-QKD system. Here, we focus on the secret key rate of the system under one-mode collective Gaussian attack, where reverse reconciliation is performed by Bob. It is important to note that the one-mode attack is not the optimal strategy. At present, the two-mode attack has been proven to be optimal. To be specific, the correlated two-mode coherent Gaussian attack are performed on two quantum channels, where the interactions of the two channels are used by Eve. However, in practical CV-MDI-QKD systems, the above correlation can become very weak when these channels come from different directions. Therefore, to facilitate analysis, the quantum channels of CV-MDI-QKD can be reduced to one-mode channel, where the one-mode attack can be efficiently performed. In particular, this simplification does not affect the results of the analysis of this article.

According to Ref. [45], the CV-MDI-QKD protocols are equivalent to the one-way CVQKD schemes using coherent states and heterodyne detection when the EPR states prepared by Bob and the displacement operation are assumed to be untrusted, which indicates that the calculation of the secret key rate of CV-MDI-QKD is the same with the standard one-way GMCS CVQKD. In the following analysis, the heterodyne detection is assumed to be perfect, and the finite-size effect is not considered. First, the Shannon mutual information between Alice and Bob can be calculated as [45,46,48]

(10) $$\begin{array}{cc} I_{AB}^{het} & =2\times \frac{1}{2}\log_{2}\frac{V_{B_{m}}^{het}}{V_{B_{m}|A_{m}}^{het}}\\ \; & =\log_{2}\frac{T_{m}\left(V_{A}+1+\chi_{\mathrm{line},m}\right)+1}{T_{m}\left(1+\chi_{\mathrm{line},m}\right)+1}, \end{array}$$

where

(11) $$\begin{array}{cc} V_{A_{m}}^{het} & =V_{A}/2+1,\\ V_{B_{m}}^{het} & =[T_{m}\left(V_{A}+1+\chi_{\mathrm{line},m}\right)+1]/2,\\ V_{B_{m}|A_{m}}^{het} & =V_{B_{m}}^{het}-\frac{T_{m}\left[{\left(V_{A}+1\right)}^{2}-1\right]}{V_{A_{m}}^{het}}\\ \; & =[T_{m}\left(1+\chi_{\mathrm{line},m}\right)+1]/2,\\ \chi_{\mathrm{line},m} & =1/T_{m}-1+\varepsilon_{m}. \end{array}$$

Then, the covariance matrix $\Gamma_{AB}^{m}$ between Alice and Bob can be written as

(12) $$\Gamma_{AB}^{m}=\left[\begin{array}{cc} aI & b\sigma_{Z}\\ b\sigma_{Z} & cI \end{array}\right],$$

where

(13) $$\begin{array}{cc} a & =V_{A}+1,\\ b & =\sqrt{T_{m}\left[{\left(V_{A}+1\right)}^{2}-1\right]},\\ c & =T_{m}V_{A}+1+T_{m}\varepsilon_{m}. \end{array}$$

Here,

(14) $$\begin{array}{cc} T_{m}= & \frac{T_{AC}}{2}k^{2},\\ \varepsilon_{m}= & 1+\frac{1}{T_{AC}}\left[2+T_{BC}\left(\varepsilon_{BC}-2\right)+T_{AC}\left(\varepsilon_{BC}-1\right)\right]\\ \; & +\frac{1}{T_{AC}}{\left(\frac{\sqrt{2}}{k}\sqrt{V_{B}}-\sqrt{T_{BC}}\sqrt{V_{B}+2}\right)}^{2}. \end{array}$$

In particular, $k=\sqrt{\frac{2V_{B}}{T_{BC}\left(V_{B}+2\right)}}$ is adopted to minimize $\varepsilon_{m}$. Based on this condition, we can obtain

(15) $$\begin{array}{cc} T_{m} & =\frac{T_{AC}V_{B}}{T_{BC}\left(V_{B}+2\right)},\\ \varepsilon_{m} & =\frac{T_{BC}}{T_{AC}}\left(\varepsilon_{BC}-2\right)+\varepsilon_{AC}+\frac{2}{T_{AC}}. \end{array}$$

In the following simulation analysis, these above channel parameters should be replaced by the estimated values in Equations (7) or (9). Then, the Holevo bound can be calculated as

(16) $$\chi_{BE}=G\left(\frac{\lambda_{m,1}-1}{2}\right)+G\left(\frac{\lambda_{m,2}-1}{2}\right)-G\left(\frac{\lambda_{m,3}-1}{2}\right).$$

Here,

(17) $$\begin{array}{cc} \lambda_{m,1,2}^{2} & =\frac{1}{2}\left(A_{m}\pm \sqrt{A_{m}^{2}-4B_{m}}\right),\\ \lambda_{m,3} & =\frac{\left(T_{m}\varepsilon_{m}+2\right)\left(V_{A}+1\right)-T_{m}V_{A}}{T_{m}\left(\varepsilon_{m}+V_{A}\right)+2}, \end{array}$$

where

(18) $$\begin{array}{cc} A_{m}= & {\left(V_{A}+1\right)}^{2}-2T_{m}\left(V_{A}^{2}+2V_{A}\right)+(T_{m}V_{A}\\ \; & +T_{m}\varepsilon_{m}{+1)}^{2},\\ B_{m}= & {\left[\left(T_{m}\varepsilon_{m}+1\right)\left(V_{A}+1\right)-T_{m}V_{A}\right]}^{2}. \end{array}$$

Finally, the secret key rate of the system can be acquired as

(19) $$K_{m}=\beta I_{AB}^{het}-\chi_{BE}.$$

Based on Equations (7), (9)–(11) and (15)–(19), the secret key rate of a CV-MDI-QKD system can be analyzed when the channel transmittance obeys the two-point distribution or the uniform distribution.

**Figure 4:**
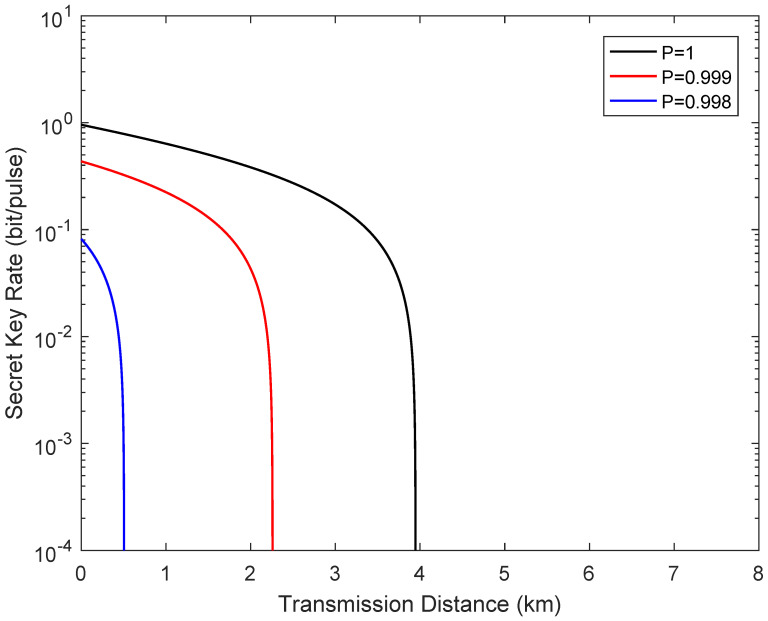
Secret key rate as a function of the transmission distance from Alice to Bob in the symmetric case when the channel transmittance obeys the two-point distribution, where $L_{AC}=L_{BC}$. The fiber loss is 0.2 dB/km.

Figure 4 describes the secret key rate versus transmission distance in the symmetric case when the channel transmittance obeys the two-point distribution. Here, the fixed parameters for the simulation are set as β=0.95, $V_{A}=V_{B}=40$, and $\varepsilon_{AC}=\varepsilon_{BC}=0.05$. The simulation results show that the fluctuating channel make the performance of the system dramatically, where P=1 represents the ideal case. It is important to note that even though the secure transmission distance is limited compared with a standard one-way CVQKD system, the demand of high-efficiency homodyne detection is removed.

**Figure 5:**
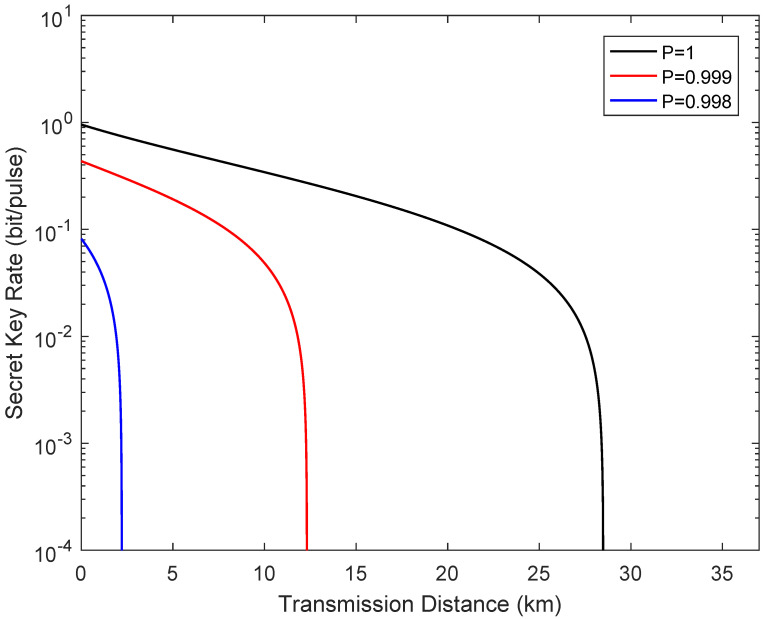
Secret key rate vs the transmission distance from Alice to Bob in the extreme asymmetric case when the channel transmittance obeys the two-point distribution, where $L_{BC}=0$.

Figure 5 reveals the secret key rate of the system as a function of the transmission distance from Alice to Bob in the extreme asymmetric case when the channel transmittance obeys the two-point distribution. The fixed parameters for simulation are the same as the symmetric case. It is obvious that the performance of the system also deteriorate under the effects of the fluctuating channel transmittance. In particular, the deterioration in the extreme asymmetric case is even worse than the symmetric case.

**Figure 6:**
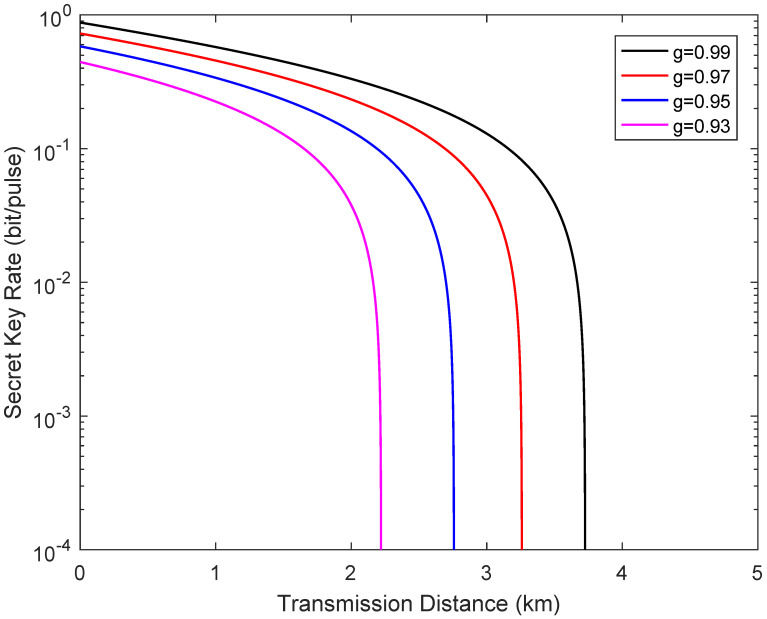
Secret key rate as a function of the transmission distance from Alice to Bob in the symmetric case when the channel transmittance obeys the uniform distribution, where $L_{AC}=L_{BC}$.

Figure 6 shows the secret key rate of the system versus transmission distance in the symmetric case when the channel transmittance obeys the uniform distribution, where *g* reflects the degree of channel jitter. Here, the fixed simulation parameters remain unchanged. We observe that the deterioration of the performance of the system increases with the degree of channel jitter.

**Figure 7:**
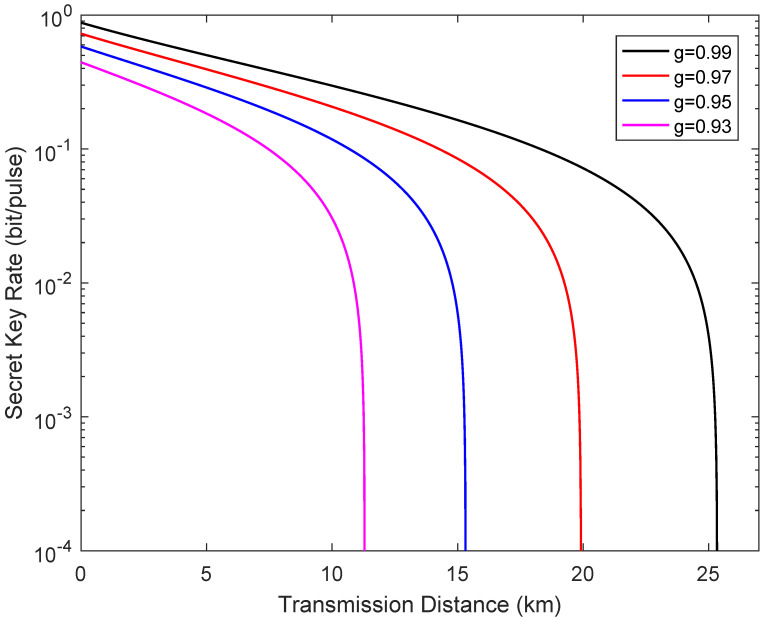
Secret key rate vs the transmission distance from Alice to Bob in the extreme asymmetric case when the channel transmittance obeys the uniform distribution, where $L_{BC}=0$.

Figure 7 depicts the secret key rate of the system as a function of the transmission distance from Alice to Bob in the extreme asymmetric case when the channel transmittance obeys the uniform distribution. The fixed parameters for simulation analysis also remain unchanged. It is clear that the dynamic trend of the performance of the system is consistent with the results shown in Figure 5.

These above simulation analyses indicate that the fluctuating channel transmittance may introduce an extra excess noise that can seriously deteriorate the performance of the practical CV-MDI-QKD systems. Correspondingly, the communication service between Alice, Bob and Charlie may be interrupted. Therefore, in a practical CV-MDI QKD systems, the potential Eve can launch a denial-service attack by manipulating the channel transmittance. To resist this attack, the Gaussian post-selection technology can be used to effectively improve the performance of the system. Specifically, Charlie first judge whether the $x_{A^{\prime }}$ and $x_{B^{\prime }}$ meet the Gaussian distribution. If the channel transmittance is manipulated, the normal linear model of the channel is destroyed. Therefore, Charlie can then extract a set of (almost) Gaussian-distributed data among the raw measurement data to calibrate the estimated values of these channel parameters to improve the performance of the system [35,57]. For example, if the channel transmittance obeys the two-point distribution, Charlie can first filter out the data when the transmittance is zero, and then complete parameter estimation. If the channel transmittance obeys the uniform distribution, Charlie can extract a set of Gaussian-distributed data when the transmittance is the low bound $gT_{0}$ to complete parameter estimation [57].

## 4. Conclusions

We have investigated the security of a practical CV-MDI-QKD system under the effects of the fluctuating channel transmittance caused by complex communication environments. We first model the fluctuating channel transmittance based on the EB scheme, and revel the deviation of parameter estimation between the fluctuating channel case and the ideal case. Furthermore, we show the parameter estimation when the channel transmittance respectively obey the two-point distribution and the uniform distribution. Based on the estimated parameters, we analyze the practical performance of the system. We observe that there is an obvious decline for the performance of the system under the impact of the fluctuating channel transmittance, especially in the extreme asymmetric case. The simulation results indicate that the fluctuating channel transmittance can produce an extra excess noise to deteriorate the system performance, which may interrupt the communication service between Alice, Bob and Charlie. This impact is more profound in the extreme asymmetric case. Therefore, a denial-service attack can be launched by Eve through manipulating the channel transmittance. To prevent this attack, the Gaussian post-selection technology is exploited to improve the performance of the system.

## Data Availability

Not applicable.
